# Independent Clinical Impacts of Procedural Complexity on Ischemic and Bleeding Events in Patients with Acute Myocardial Infarction: Long-Term Clinical Study

**DOI:** 10.3390/jcm11164853

**Published:** 2022-08-18

**Authors:** Kwan Yong Lee, Byung-Hee Hwang, Sungmin Lim, Chan Jun Kim, Eun-Ho Choo, Seung Hoon Lee, Jin-Jin Kim, Ik Jun Choi, Gyu Chul Oh, In-Ho Yang, Ki Dong Yoo, Wook Sung Chung, Youngkeun Ahn, Myung Ho Jeong, Kiyuk Chang

**Affiliations:** 1 Cardiology Division, Cardiovascular Center, Seoul St. Mary’s Hospital, The Catholic University of Korea, Seoul 06591, Korea; 2 Cardiology Division, Cardiovascular Center, Uijeongbu St. Mary’s Hospital, The Catholic University of Korea, Uijeonbu 11765, Korea; 3 Cardiology Division, Cardiovascular Center, Incheon St. Mary’s Hospital, The Catholic University of Korea, Incheon 21431, Korea; 4 Department of Cardiovascular Medicine, Kyung Hee University Hospital, Seoul 05278, Korea; 5 Cardiology Division, Cardiovascular Center, St. Vincent’s Hospital, The Catholic University of Korea, Suwon 16247, Korea; 6 Department of Cardiology, Cardiovascular Center, Chonnam National University Hospital, Gwangju 61469, Korea

**Keywords:** complex percutaneous coronary intervention, antiplatelet therapy, acute myocardial infarction, drug-eluting stents, risk factor

## Abstract

This study aimed to investigate the relationship between a complex percutaneous coronary intervention (C-PCI) and long-term clinical outcomes in the AMI cohort. A total of 10,329 patients were categorized into the C-PCI and non-C-PCI groups. The primary ischemic endpoint was a composite of major adverse cardiac events (MACEs, cardiac death, myocardial infarction, stent thrombosis and revascularization). The primary bleeding endpoint was the risk of overt bleeding (BARC 2, 3 or 5). The median follow-up duration was 4.9 (2.97, 7.16) years. The risks of MACEs and bleeding were significantly higher in the C-PCI group (hazard ratio (HR): 1.72; 95% confidence interval (CI): 1.60 to 1.85; *p* < 0.001; and HR: 1.32; 95% CI: 1.17 to 1.50; *p* < 0.001, respectively). After propensity score matching, compared to the non-C-PCI group, the adjusted MACE rate in C-PCI remained significantly higher (*p* < 0.001), but no significant interaction (*p* = 0.273) was observed for bleeding. Significant differences in overt bleeding were observed only within the first three months (*p* = 0.024). The MACEs were consistently higher in the C-PCI group with or without severe comorbid conditions (*p* < 0.001 for both). Patients with AMI who undergo C-PCI experience worse long-term ischemic outcomes after successful PCI, regardless of the presence of severe comorbidities.

## 1. Introduction

Percutaneous coronary intervention (PCI) techniques and drug-eluting stents have evolved over the past 40 years, and they are now relatively safe and effective [1]. However, PCI in patients with more complex lesions is still associated with periprocedural complications and high rates of restenosis [2,3]. Currently, approximately 40% of PCIs are considered complex PCIs, although further clarity is needed on the definition of complex PCI procedures [4,5]. Verification of whether complex PCI impacts poor prognosis in various clinical presentations is also needed. Proper categorization of anatomical and procedural profiles accompanying poor ischemic outcomes might affect the decision when it comes to determining the duration and intensity of the dual antiplatelet strategy [6,7,8]. Extending DAPT may reduce this risk; however, current guidelines recommend DAPT duration only according to the clinical manifestation of disease (e.g., stable coronary artery disease, non-ST-segment elevation acute coronary syndrome or ST-segment elevation myocardial infarction (STEMI)) [9]. Even in STEMI patients, the use of DAPT beyond one year is not strongly recommended. Although previous studies have suggested that complex PCI has a poor prognosis and long DAPT use will help, it is still controversial whether it is acceptable to determine a DAPT treatment strategy solely on procedural complexity [10,11,12]. In previous studies, the influence of procedural complexity has not been independently analyzed, and baseline comorbidities (e.g., old age, chronic kidney disease, reduced left ventricular ejection fraction) in patients who underwent complex PCI may have contributed to poor prognosis [13,14,15]. This study aimed to evaluate the independent influence of complex PCI on long-term prognosis within the acute myocardial infarction (AMI) cohort, which is already considered to have a high current ischemic event incidence. The influence of baseline comorbid conditions in patients who can act as confounding variables was eliminated through the propensity score (PS) matching method and through subgroup analysis divided by the presence of comorbidities.

## 2. Materials and Methods

### 2.1. Study Protocols and Population Selection

**Figure 1:**
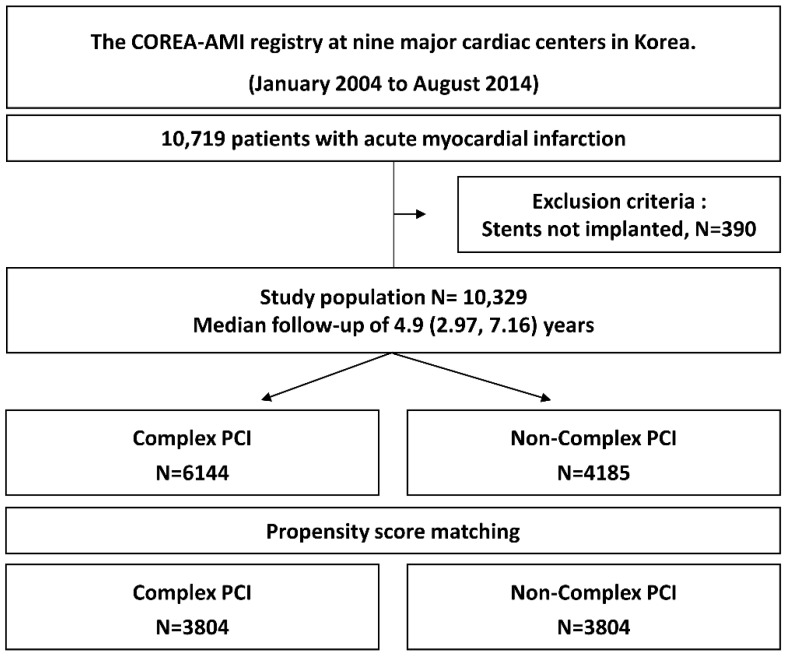
Study flow.

The COnvergent REgistry of cAtholic and chonnAm University for Acute MI (COREA-AMI) registry was designed to evaluate real-world, long-term clinical outcomes in all consecutive patients with AMI at nine major cardiac centers in Korea. All hospitals perform high-volume PCI in AMI patients and are located throughout the country. The COREA-AMI I registry included AMI patients undergoing PCI from January 2004 to December 2009, and the COREA-AMI II registry extended the follow-up period of COREA-AMI I patients and enrolled additional AMI patients from January 2010 to August 2014. Clinical, angiographic and follow-up data of all AMI patients were consecutively registered in the electronic, web-based case report system. The COREA-AMI study was conducted in accordance with the Declaration of Helsinki. This observational study was approved by the institutional review board of our institution and performed in accordance with Strengthening the Reporting of Observational Studies in Epidemiology guidelines [16]. The COREA-AMI registry is registered on ClinicalTrials.gov (study ID: NCT02806102). In total, 10,719 patients with AMI who received PCI using drug-eluting stents were enrolled in the registry, and 390 patients who did not undergo PCI were excluded from the analysis. Thus, 10,329 patients were selected for this analysis. A study flowchart is depicted in Figure 1. The objective of the present study was to evaluate the effect of procedural complexity on ischemic and bleeding clinical outcomes following revascularized AMI. All participating patients were divided into a complex PCI group and a non-complex PCI group.

### 2.2. Definitions

Complex PCI was defined as a procedure with at least one of the following angiographic characteristics: the left main as the target vessel, bifurcation PCI with two stents, multivessel PCI, >60 mm long stent implantation, restenosis lesion, CTO lesion, ≥3 treated lesions or ≥3 stents implantation. Each complex PCI criterion had to have at least one angiographic feature associated with an increased risk of ischemic events based on previous reports and guidelines [17,18,19,20,21,22]. AMI was diagnosed by characteristic clinical symptoms, serial changes in ECGs consistent with infarction, and elevated cardiac enzyme values. The diagnosis was confirmed by coronary angiography in all patients.

### 2.3. PCI Procedure and Medical Treatment

All patients underwent PCI within 48 h after admission. Coronary angiography and primary PCI were performed according to the current standard guidelines. The significant coronary disease was defined by angiographic stenosis ≥70% in the epicardial coronary arteries and ≥50% in the left main coronary artery. The loading dose of the antiplatelet agent (aspirin, 300 mg; clopidogrel, 300 mg or 600 mg; cilostazol, 200 mg; ticagrelor, 180 mg; or prasugrel, 60 mg) was prescribed for all patients before or during PCI. Patients with drug-eluting stents (DESs) were prescribed P2Y12 inhibitors (clopidogrel, 75 mg once daily; ticagrelor, 90 mg twice daily; or prasugrel, 10 mg once daily) and/or aspirin, 100 mg daily. The duration of dual antiplatelet agent administration was determined by a physician in accordance with the final diagnosis at baseline and the revascularization procedure complexity. Optimal pharmacological therapy, including statins, beta-blockers, angiotensin-converting enzyme (ACE) inhibitors or angiotensin II receptor blockers (ARBs) was recommended according to the guidelines. Doses were titrated, and medications were changed during follow-up if needed due to each patient’s condition. Predilatation, direct stenting, postadjunct balloon inflation and glycoprotein IIb/IIIa receptor blocker administration were performed at the discretion of individual physicians.

### 2.4. Study Endpoints and Follow-Up

The primary ischemic endpoint of this analysis was the composite of major adverse cardiac events (MACE: composite of cardiac death, recurrent myocardial infarction, definite or probable stent thrombosis, or any repeat revascularization) five years after AMI index PCI. The secondary ischemic outcomes were all-cause death, cardiac death, recurrent MI, stroke, any revascularization, target vessel revascularization (TVR), target lesion revascularization (TLR) and stroke. The primary bleeding endpoint was overt bleeding (Bleeding Academic Research Consortium (BARC) type 2, 3 or 5) [23]. Secondary bleeding endpoints included BARC types 3 and 5 or any bleeding. Clinical outcomes within three months and after three months from index PCI were also compared between the two groups. All deaths were considered cardiac unless an undisputed non-cardiac cause was present. Recurrent MI was defined as the presence of recurrent symptoms and new ECG changes that were compatible with MI, or cardiac markers that were expressed at least twofold above the normal limit. Clinically driven revascularization that occurred after discharge from the index hospitalization was coded as a repeat revascularization event, according to the Academic Research Consortium definition. Stroke was defined as the presence of a new focal neurologic deficit thought to be vascular in origin, with signs or symptoms lasting more than 24 h. Each patient was followed up at outpatient clinics or by telephone questionnaire at 1, 6 and 12 months, and then annually thereafter. Information related to censored survival data (death or survival) and cause of death (cardiac or non-cardiac death) was confirmed with the Korean Office of Statistics (KOSTAT) with a unique personal identification number to validate the complete follow-up data. All clinical outcomes of interest were confirmed by source documents and centrally adjudicated by a clinical events committee at the Cardiovascular Center of Seoul St Mary’s Hospital, which consisted of an independent group of clinicians whose members were unaware of patient status.

### 2.5. Statistical Analysis

Categorical variables were presented as numbers and relative frequencies (percentages) and were compared using the Chi-squared test or Fisher’s exact test. Continuous variables were expressed as mean ± standard deviation or median (Q1, Q3), according to whether they were normally distributed or not, and were compared using the independent sample *t*-test or Mann-Whitney U test, as appropriate. The cumulative ischemic and bleeding event rates of each group (complex PCI vs. non-complex PCI) were calculated using a Kaplan-Meier estimator and compared using the log-rank statistic. Unadjusted hazard ratios for five-year outcomes were determined from Cox proportional hazards models. Because differences in baseline characteristics could significantly affect outcomes, sensitivity analyses were performed to adjust for confounders as much as possible. First, a multivariable Cox proportional hazard regression model was used. The adjusted variables for the multivariate model were selected if they were significantly different between the two groups (showing a *p*-value of <0.05 in univariable analysis) at baseline data, including clinical characteristics, laboratory tests, medications at discharge and extended DAPT usage (Table 1). Second, Cox proportional hazard regression in a PS matched cohort was performed. Propensity scores were obtained from logistic regression with a significant difference between the two groups. We employed nearest-neighbor matching using a caliper of 0.2 multiplied by the standard deviation for linearly transformed propensity scores. PS matching yielded 2636 patients in the complex PCI group and 2636 control subjects in the non-complex PCI group. The balance between the two groups after PS matching was assessed by calculating percent standardized mean differences. The percent standardized mean differences after PS matching were within ±10% across all matched covariates, demonstrating successful balance achievement between comparative groups (Table 1). Third, consistency of the effect of complex PCI according to the presence of severe comorbidities was evaluated by repeated Cox analysis at each subgroup. Any of the following characteristics was defined as severe comorbidities: old age, diabetes mellitus with insulin treatment, prior MI, prior CABG, established vascular disease, atrial fibrillation, chronic kidney disease, severe left ventricle dysfunction, cardiogenic shock and ECMO/IABP usage. Each measure was analyzed using SAS 9.4 (SAS Institute, Cary, NC, USA). Statistical significance was indicated by a two-tailed *p* < 0.05.

## 3. Results

### 3.1. Baseline Patient Characteristics

The baseline clinical, angiographic and procedural characteristics are listed in Table 1 and Table 2. The mean age of all included patients was 63.6 ± 12.8 years. The median follow-up duration was 4.9 (2.97, 7.16) years. Of the 10,329 included patients, 6144 patients underwent complex PCI and 4185 underwent non-complex PCI. Overall, 36.7% had diabetes mellitus, 70.1% had hypertension, 6.0% had chronic kidney disease (estimated glomerular filtration rate, eGFR < 30) and 3.6% had previous MI. Regarding angiographical lesion and procedural profiles, 12.1% of patients presented with cardiogenic shock and 6.2% required hemodynamic support device use. Patients with complex PCI were more likely to be older, have a current smoking habit and have diabetes mellitus, high blood pressure, chronic kidney disease, history of MI, PCI and CABG, KILLIP III or IV, cardiogenic shock, non-ST segment elevation MI, a low left ventricular ejection fraction, high peak troponin I and CK-MB levels and a high GRACE score.

### 3.2. Baseline Lesion and Procedure-Related Characteristics (Propensity Score-Matched Cohort)

**Figure 2:**
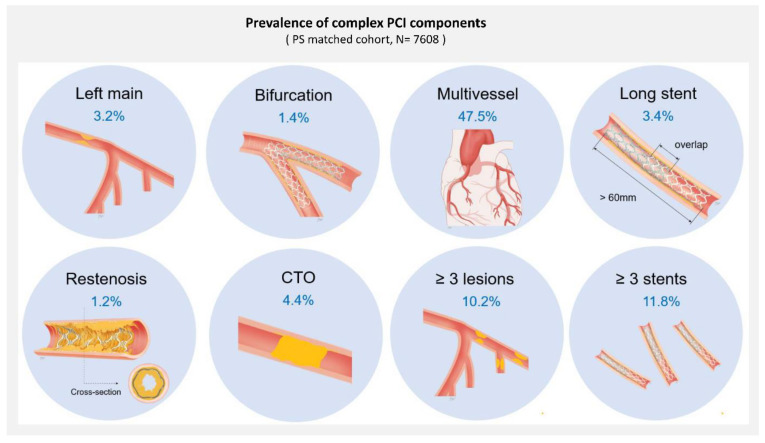
The definition of complex PCI and prevalence of each component in the PS matched cohort.

Most of the implanted stents in both the complex and non-complex PCI groups were second-generation drug-eluting stents (68.0% and 67.8%, respectively) (Table 2). A mean of 1.9 ± 1.0 stents was implanted in the complex PCI group compared to 1.2 ± 0.4 in the non-complex PCI group. The total stent length of the complex PCI group was 40.9 ± 23.4 mm, and that of the non-complex PCI group was 23.9 ± 8.4 mm. Among the total of 7608 patients from the PS matched cohort, the proportions of left main artery stenting, bifurcation PCI with two stents, multivessel PCI, >60 mm long stent implantation, restenosis lesion, CTO lesion, ≥3 lesions treatment or ≥3 stents implantation were 3.2%, 1.4%, 47.5%, 3.4%, 1.2%, 4.4%, 10.2% and 11.8%, respectively (Figure 2).

### 3.3. Independent Predictors of the Primary Ischemic Endpoint

**Figure 3:**
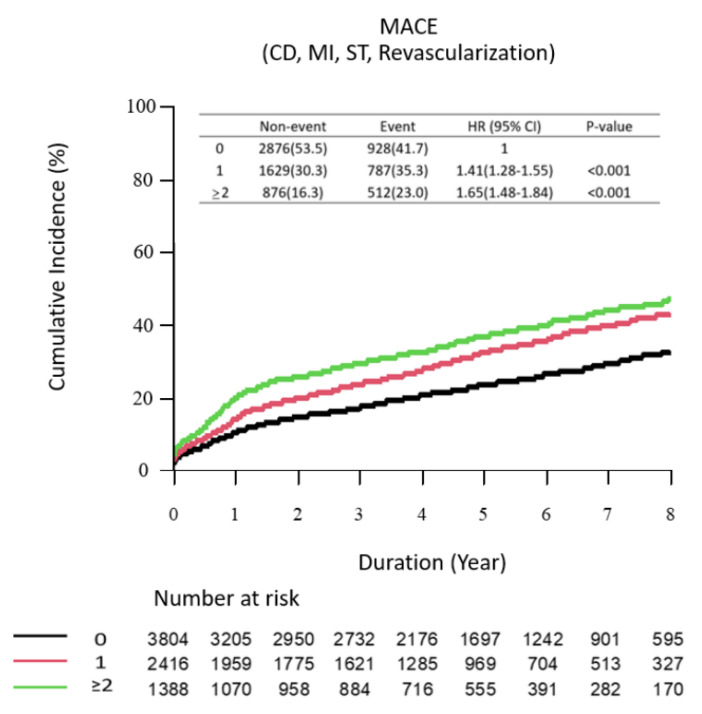
Comparison of the K-M Curve According to the Number of Complex PCI Variables. Cumulative incidence of MACEs in the PS-matched cohort stratified by the number of complex PCI components. MACE is defined as the composite of CD, MI, ST or revascularization. CD indicates cardiac death, MI—myocardial infarction, ST—definite or probable stent thrombosis.

The prevalence and hazard risks of the individual qualifying variables within the complex PCI group are described in (Figure 2 and Table 3). Univariate and multivariate Cox proportional hazard models and PS matching were performed. By including each component of complex PCI as a variable within the same Cox analysis models separately, seven components were independent predictors of MACEs after the procedure (*p* < 0.05, each), while bifurcation with two stents at non-LM artery showed no significant association with the risk of MACEs (*p* = 0.108). The hazard models also identified independent clinical predictors (e.g., old age, diabetes mellitus with insulin treatment, prior MI, prior CABG, established vascular disease, atrial fibrillation, chronic kidney disease, severe left ventricle dysfunction, cardiogenic shock and ECMO/IABP usage) of the primary ischemic endpoint. Complex PCI remained strongly associated with an increased risk of MACEs; however, the magnitude was numerically lower than that of severe clinical comorbidities such as old age, prior CABG, chronic kidney disease, severe LV dysfunction, cardiogenic shock or hemodynamic support device usage [21,24]. By including complex PCI as a continuous variable (per increase in the number of complex PCI variables) within the Kaplan-Meier estimator, the risk of MACE events significantly increased as the number of high-risk procedural characteristics increased (Figure 3).

### 3.4. Ischemic Outcomes According to PCI Complexity

**Figure 4:**
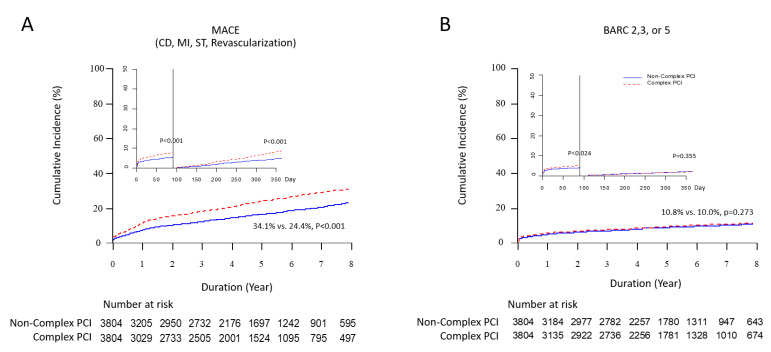
Ischemic and Bleeding Outcomes in AMI Patients According to PCI Complexity. Kaplan-Meier curves with cumulative hazards of (**A**) MACE (CD, MI, ST and revascularization) and (**B**) BARC 2, 3 or 5 bleeding compared according to the procedural complexity in the propensity score matched cohort.

The Kaplan-Meier survival curve for clinical events, according to procedural complexity in the PS-matched cohort, is shown in Figure 4. A comparison of ischemic and bleeding clinical outcomes between the complex and non-complex PCI groups is presented in Table 4. The risk of MACEs was significantly higher in the complex PCI group than in the non-complex PCI group (37.8% vs. 23.8%; hazard ratio (HR): 1.72, 95% confidence interval (CI): 1.60 to 1.85, *p* < 0.001) and was driven by a significantly higher risk of cardiac death, myocardial infarction, stent thrombosis and revascularization. Sensitivity analyses using multivariable Cox regression and PS matching consistently showed significantly higher risks of MACEs, cardiac death, myocardial infarction, stent thrombosis, revascularization and TVR in the complex PCI group than in the non-complex PCI group. However, the differences among all-cause death, ischemic stroke and TLR were no longer significant after PS matching. Interesting findings were observed from the three-month and one-year landmark analyses. Stent thrombosis, TVR and TLR occurred more frequently in the complex PCI group until three months after index PCI; however, the differences became non-significant beyond three months (Table S1 and S2). In addition, there were no significant differences in mortality or cardiac death between the two groups before one year; however, a significantly higher risk was observed after one year (Tables S1 and S3). A total of 55.3% all patients were prescribed extended DAPT over one year. There was no significant difference in extended dual antiplatelet usage over one year in the complex PCI and non-complex PCI groups (56.6% vs. 54.9%, *p* = 0.137) (Table 1).

### 3.5. Bleeding Outcomes According to PCI Complexity

**Figure 5:**
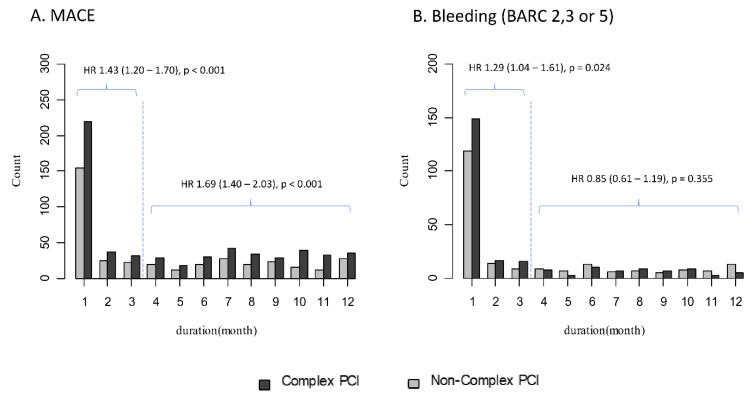
Cumulative incidence of ischemic (**A**) and bleeding (**B**) outcomes according to PCI complexity at each month until a follow-up duration of one year (three-month landmark analysis data in the PS matched cohort).

In Table 4, the risk of BARC 2, 3 or 5 bleeding was significantly higher in the complex PCI group (12.0% vs. 9.7%; HR: 1.26, 95% CI: 1.12–1.42, *p* < 0.001). On the other hand, it became non-significant after multivariable adjustment or PS matching. Interestingly, during the initial three months, the risk of BARC 2, 3 or 5 bleeding was significantly higher in the complex PCI group, even in the PS-matched cohort (Figure 5 and Table S1). Beyond three months, there was no difference in overt bleeding between the two groups (Figure 4B and Table S2). In our cohort, there was no significant difference in the number of patients prescribed potent P2Y12 inhibitors (ticagrelor or prasugrel) at discharge or in the number of patients who continued to use the drug for one year between the complex PCI group and the non-complex PCI group (12.6% vs. 13.3%, *p* = 0.341; 9.9% vs. 10.8%, *p* = 0.216, respectively) (Table 1). Among patients with BARC 2, 3 or 5 bleeding events within a year, the number of patients prescribed potent P2Y12 inhibitors as discharge medication and the number of patients who were still using the drug at one year was higher than those who were not, but there were no significant differences (13.7% vs. 12.5%, *p* = 0.190; 11.9% vs. 10.5%, *p* = 0.249). In addition, there was no significant difference in extended dual antiplatelet usage over one year in the complex PCI and non-complex PCI groups (56.6% vs. 54.9%, *p* = 0.137).

### 3.6. Effects of Complex PCI According to the Presence of Clinical Comorbidities

The effects of complex PCI on clinical ischemic outcomes in patients without comorbid conditions are presented in Table 5. The risk of MACEs was higher in the complex PCI group than in the non-complex PCI group (26.9% vs. 15.9%; HR: 1.82, 95% CI: 1.60–2.08, *p* < 0.001). The difference was mainly driven by MI, stent thrombosis and revascularization. In contrast with the ischemic outcomes, there were no statistically significant differences between the complex PCI and non-complex PCI in BARC class 2 or higher bleeding (7.9% vs. 7.3%; HR: 1.09, 95% CI: 0.89–1.34, *p* = 0.407). The effects of complex PCI in patients with severe comorbid conditions showed a consistent trend with that in patients without comorbid conditions. For both ischemic and bleeding endpoints, no interactions were present when stratified by the presence of comorbidities.

## 4. Discussion

In the present study, we compared five-year clinical outcomes of complex PCI versus non-complex PCI in patients with AMI using data from a large multicenter observational study. The main findings were as follows. First, we used a recently accepted definition of complex PCI from the 2020 ESC NSTEMI guideline [19] and verified that each type of complex procedure was independently associated with higher rates of ischemic events in the AMI cohort (Figure 2 and Table 4). Second, the complex PCI group showed a significantly higher risk of MACEs than the non-complex PCI group during the entire follow-up period (Figure 4A). The results remained consistent after multivariable regression and PS matching to adjust for baseline differences (Table 4). The primary ischemic outcome was mainly driven by cardiac death, stent thrombosis and revascularization. Third, within three months following index PCI, overt bleeding occurred significantly more frequently in the complex PCI group than in the non-complex PCI group (Figure 5). The difference was not observed after the initial three months. Fourth, significantly higher risks of MACEs in the complex PCI group than in the non-complex PCI group were consistently observed in subgroups with or without severe comorbidities (Table 3 and Table 5).

In our cohort, we defined complex PCI with components widely known as a high thrombotic risk in the previous reports and in the recent ESC guidelines of 2020 [18,19,25,26] (Figure 2). A total of 59.5% of the AMI patients who underwent PCI met our criteria for complex PCI. In particular, multivessel PCI had a high proportion, but of course, this ratio reflects the duplication of other components. Eight components of complex intervention procedures, with the left main as the target vessel, bifurcation PCI with two stents, multivessel PCI, >60 mm long stent implantation, restenosis, CTO lesion, ≥3 lesions treated and ≥3 stents implanted, were independently associated with poor major ischemic events. Some studies have addressed the association between each complex PCI component and hard clinical endpoints, such as cardiovascular mortality or myocardial infarction; however, data from the AMI population are scarce [2,27]. In our study, the presence of procedural complexity significantly affected poor prognosis in AMI patients successfully revascularized with coronary stents (second-generation drug-eluting stent usage: 65.8%). A large stent strut burden at the carina or overlap of multiple stent layers might increase this risk due to increased wall shear stress and platelet aggregation promotion [11]. In our multivariable model, each type of complex procedure was independently associated with higher rates of ischemic events than others (Table 3). The greater the risk of repeated ischemic events, the greater the number of procedural complexities present (Figure 3). In previous studies, patients who underwent complex PCI showed poor prognosis, but did not see the independent influence of procedural complexity [2,3]. It has been explained that the baseline comorbidity of patients with complex PCI also contributed to poor prognosis [13,14,15]. These characteristics are associated with a wider range of disease burden and lesion complexity, with more stent insertion, platelet activation and ischemic clinical events [6,28]. In this study, the independent influence of complex PCI was evaluated within the AMI cohort, which is already thought to have a high recurrent ischemic event risk. Comorbidity, medication, DAPT duration, etc., were adjusted through PS matching. We additionally verified the influence of procedural complexity on prognosis in subgroups without or without severe comorbidity. The results consistently showed that the ischemic outcomes were worse than those of the non-complex PCI group in the complex PCI group (Table 4).

Patients with complex PCI are at high risk of ischemic clinical events, and this risk may be reduced by extending DAPT versus aspirin alone [3,6,8]. However, the preferred DAPT duration for patients who undergo complex PCI remains a matter of debate. In the current guidelines, DAPT duration is recommended according to clinical manifestation, and is recommended for use for one year in AMI patients [19,29]. On the other hand, it is recommended to use a potent P2Y12 inhibitor in the early stages of AMI for patients with complex PCI, but the guidance for a long DAPT duration is unclear. Previous randomized trials of longer DAPT durations beyond 1 year (DAPT and PEGASUS-TIMI54) have found that longer DAPT durations lead to fewer ischemic events at the expense of greater bleeding events [25,26]. Some registry studies or meta-analysis results have also suggested a benefit of extended DAPT usage over one year [30,31,32]. The decision to extend the DAPT duration in specific clinical scenarios depends on individual clinical judgments driven by the patient’s risk of ischemia and bleeding, adverse events and comorbidities. Patients with a high risk of bleeding can be defined as patients with a PRECISE-DAPT score of 25 or higher or meeting the ARC-HBR criteria, but patients with a high risk of ischemia do not have a useful scaling method in practice [24]. Although the DAPT score was proposed as a criterion for determining whether to use DAPT for 12 months vs. 30 months, the high ischemic risk list contained few components of procedural complexity, and the procedure-related environment changed compared to when the trial was conducted [33]. Performance and technologies (e.g., thickness, material, released drugs, etc.) of coronary intervention have improved; however, the proportion of high-risk procedures such as LM PCI or bifurcation PCI has increased over time [34]. In addition, a domain such as age is a high risk factor for ischemia and, simultaneously, a high risk factor for bleeding. Therefore, there is a limit to applying DAPT scores to real-world clinical practice in elderly patients [35]. In addition, there is little information on whether DAPT should be maintained for longer, over three years, in the ischemic high-risk patient group.

In previous reports, major bleeding incidences also increased among patients who underwent complex PCI and have been associated with higher mortality, underlying the challenge of treating such patients [36,37,38]. In our data, bleeding events occurred more frequently in the complex PCI group only within the initial three-month follow-up period. BARC 2, 3 or 5 bleeding risks exhibited 1.29-fold increases during this period (*p* = 0.024) (Table S1A). In our study, in-hospital bleeding events occurred in 386 (3.7%) patients. Among them, puncture site bleeding occurred in 139 (36.0% of in-hospital bleeding cases). The predictors of in-hospital bleeding were complex PCI, old age, female, diabetes mellitus, low eGFR (<30), history of stroke, low LVEF (≤35%) and ECMO/IABP use (Table S4). After PS matching, the difference of in-hospital bleeding rate between complex and non-complex PCI changed from significant (unadjusted HR: 1.46, 95% CI: 1.18–1.80, *p* < 0.001) to marginal (HR: 1.28, 95% CI: 1.00–1.63, *p* = 0.046). More heparin might be used in complex cases and may possibly cause more in-hospital bleeding; however, our cohort lacks data about heparin infusion amount and duration. Femoral access, GP IIb/IIIa inhibitor use and thrombolysis infusion did not differ between complex PCI and non-complex PCI groups. The difference in BARC 2, 3 or 5 bleeding events between complex PCI and non-complex PCI was not observed beyond three months following index PCI (HR 0.96, 95% CI: 0.80–1.14, *p* = 0.614) (Table S2). A plausible explanation for this is that potent P2Y12 inhibitors were not used more often (*p* = 0.987), and there was no significant difference in extended dual antiplatelet usage over one year in the complex PCI and non-complex PCI groups (*p* = 0.137) (Table 1). Furthermore, 65.8% of stents registered in this study were second-generation DESs. Late stent thrombosis is less likely to occur in the second-generation DES era than in the first-generation DES era [39,40], although complex PCI procedures increase the risk of stent thrombosis [41].

Our study results suggest that using a more potent P2Y12 inhibitor or maintaining a DAPT strategy may be needed for the complex PCI group due to the higher incidence of MACEs during five-year long-term follow-up periods, regardless of the baseline comorbidities of AMI patients. In addition, more attention is required during the initial three months after index PCI considering the higher rate of overt bleeding occurrence. At present, to specify a proper DAPT duration for AMI patients with complex PCI, further studies with large volume randomized tests are needed. A tailored strategy of DAPT intensification in selected patients with AMI undergoing complex PCI, considering the individualized ischemia vs. bleeding risk, may provide a net clinical benefit.

### Limitations

The first limitation of this study was that it was retrospective, which decreased the statistical power to detect differences. However, with extensive sensitivity analyses and large population cohort data, possible confounders were adjusted to minimize bias from different baseline characteristics. Second, data on the exact duration of dual antiplatelet therapy were missing. Therefore, the ability to specify the DAPT duration and identify the efficacy of the extended DAPT strategy in the complex PCI group was not available in this study. Further analysis associated with the efficacy of the extended DAPT strategy and proper duration of DAPT is needed. Third, the very long period of study could be a confounder because complex PCI could be changed due to longitudinal bias, despite careful follow-up. Although our study includes more than a majority of second-generation drug release stents, it does not represent the results of the latest contemporary stents usage. Fourth, the global standard definition has not yet been determined for each element constituting the complex PCI. In our work, we adopted the components associated with the procedure among the high thrombotic risk predictors mentioned in the latest guidelines [19]. CTO PCI was included in the complex PCI group, considering the effect of stent burden on long-term prognosis, although there was a debate on whether it was right to include it in a study of the acute MI population. Adjunctive device usage that may be associated with actual procedural difficulties, such as atherectomy devices or a cutting balloon, was excluded due to the too-low utilization rate (<0.1%) within this registry data. Additional statistical analyses were conducted by changing the definition of complex PCI, including or excluding CTO PCI, and the results were consistent. Fifth, among potential complications, contrast-induced nephropathy and its impact on prognosis were not investigated due to a lack of data on contrast volume and radiation exposure time.

## 5. Conclusions

Among patients with AMI, complex PCI—defined by a combination of high-risk angiographic and procedural features—was associated with significantly higher risks of ischemic events during long-term follow-up periods. The results remained consistent after multivariable regression, propensity score matching and subgroup analysis with/without severe comorbidities. The risk of recurrent ischemic events was more remarkable for the greater number of procedural complexities. The present study results suggest that long-term preventive antithrombotic medication strategies may be needed for patients who have undergone complex PCI, regardless of the presence of clinical comorbidities.

## Figures and Tables

**Table 1:** Baseline characteristics.

	Before PS Matching				After PS Matching			
	Complex PCI (*n* = 6144)	Non-Complex PCI (*n* = 4185)	*p*-Value	Absolute SMD	Complex PCI (*n* = 3804)	Non-Complex PCI (*n* = 3804)	*p*-Value	Absolute SMD
**Clinical characteristics**								
Age, year	65.1 ± 12.2	61.3 ± 13.3	<0.001	0.298	62.6 ± 12.3	62.5 ± 12.8	0.984	0.003
≥75	1505 (24.5)	774 (18.5)	<0.001	0.146	706 (18.6)	755 (19.8)	0.128	0.033
Female	1850 (30.1)	1083 (25.9)	<0.001	0.094	1021 (26.8)	1026 (27.0)	0.895	0.003
BMI	24.0 ± 3.1	24.3 ± 3.3	0.001	0.070	24.2 ± 3.1	24.2 ± 3.3	0.817	0.010
DM	2495 (40.6)	1293 (30.9)	<0.001	0.204	1271 (33.4)	1253 (32.9)	0.638	0.010
With insulin treatment	155 (2.5)	53 (1.3)	<0.001	0.092	3753 (98.7)	3751 (98.6)	0.841	0.005
HBP	4461 (72.6)	2783 (66.5)	<0.001	0.133	2576 (67.7)	2611 (68.6)	0.375	0.020
Dyslipidemia	1402 (22.8)	953 (22.8)	0.955	0.001	969 (25.5)	841 (22.1)	0.001	0.079
History of stroke	510 (8.3)	240 (5.7)	<0.001	0.101	239 (6.3)	234 (6.2)	0.809	0.005
Current smoker	2348 (38.2)	1828 (43.7)	<0.001	0.111	1584 (41.6)	1600 (42.1)	0.705	0.009
Previous MI	249 (4.1)	126 (3.0)	0.005	0.056	136 (3.6)	123 (3.2)	0.41	0.019
Previous PCI	415 (6.8)	196 (4.7)	<0.001	0.089	212 (5.6)	195 (5.1)	0.379	0.020
Previous CABG	34 (0.6)	13 (0.3)	0.072	0.037	18 (0.5)	13 (0.3)	0.369	0.021
Atrial fibrillation on baseline ECG	180 (2.9)	134 (3.2)	0.429	0.016	92 (2.4)	124 (3.3)	0.029	0.051
eGFR < 30	443 (7.2)	179 (4.3)	<0.001	0.126	191 (5.0)	172 (4.5)	0.289	0.023
LVEF	52.4 ± 11.3	54.4 ± 10.3	<0.001	0.182	53.9 ± 10.6	54.0 ± 10.3	0.657	0.010
LVEF ≤ 35%	533 (8.7)	201 (4.8)	<0.001	0.155	231 (6.1)	200 (5.3)	0.12	0.035
KILLIP III or IV	1019 (16.6)	541 (12.9)	<0.001	0.103	535 (14.1)	510 (13.4)	0.394	0.019
Cardiogenic shock	769 (12.5)	476 (11.4)	0.08	0.035	431 (11.3)	441 (11.6)	0.719	0.008
ST-segment elevation MI	3171 (51.6)	2490 (59.5)	<0.001	0.159	2181 (57.3)	2191 (57.6)	0.81	0.005
Troponin I, peak, ng/mL	77.6 ± 1617.3	57.8 ± 143.0	<0.001	0.017	50.1 ± 88.5	58.2 ± 149.2	0.09	0.066
CK-MB, peak, ng/mL	132.2 ± 823.5	131.3 ± 274.4	0.003	0.002	132.4 ± 519.7	131.2 ± 278.2	0.555	0.003
GRACE score	140.3 ± 44.9	131.6 ± 44.1	<0.001	0.195	135.3 ± 44.1	133.6 ± 44.1	0.213	0.038
**Medication at discharge**								
Aspirin	5690 (98.0)	3993 (98.7)	0.007	0.056	3581 (97.9)	3616 (98.6)	0.06	0.048
Clopidogrel	5066 (87.2)	3435 (84.9)	0.001	0.067	3114 (85.2)	3142 (85.6)	0.611	0.014
Ticagrelor	326 (5.6)	235 (5.8)	0.648	0.009	222 (6.1)	208 (5.7)		0.017
Prasugrel	369 (6.3)	364 (9.0)	<0.001	0.100	292 (8.0)	306 (8.3)	0.629	0.014
Beta blocker	4753 (85.2)	3409 (89.2)	<0.001	0.122	3071 (88.3)	3090 (88.4)	0.905	0.004
ACEi or ARB	4431 (72.1)	3223 (77.0)	<0.001	0.113	2883 (75.8)	2899 (76.2)	0.662	0.010
Oral anticoagulant	146 (2.4)	86 (2.1)	0.279	0.022	85 (2.2)	80 (2.1)	0.694	0.009
Statin at discharge	5221 (95.5)	3663 (96.8)	0.001	0.069	3320 (96.6)	3321 (96.6)	0.719	0.000
High-dose statin	1171 (19.1)	872 (20.8)	0.083	0.044	805 (21.2)	770 (20.2)	0.601	0.023
Moderate-dose statin	4716 (76.8)	3140 (75.0)		0.040	2834 (74.5)	2875 (75.6)		0.025
Low-dose statin	257 (4.2)	173 (4.1)		0.002	165 (4.3)	159 (4.2)		0.008
Extended DAPT (>1 year)	3364 (54.8)	2352 (56.2)	0.146	0.029	2138 (56.2)	2141 (56.3)	0.945	0.002

Data are presented as the *n* (%) for categorical variables and as the mean ± standard deviation for continuous variables. PS indicates propensity score; PCI—primary coronary intervention; SMD—standardized mean difference; BMI—body mass index; DM—diabetes mellitus; HBP—high blood pressure; MI—myocardial infarction; CABG—coronary artery bypass graft; ECG—elctrocardiography; eGFR—estimated glomerular filtration rate; LVEF—left ventricle ejection fraction; KILLIP—Killip classification, the classification of heart failure severity in patients with acute myocardial infarction; ECMO—extracorporeal membrane oxygenation; IABP—intra-aortic balloon pump; CK-MB—creatinine kinase MB isoenzyme; ACEi—angiotensin converting enzyme inhibitors; ARB—angiotensin II receptor blockers; DAPT—dual antiplatelet therapy.

**Table 2:** Baseline lesion- and procedure-related characteristics (PS matched cohort).

	Total (*n* = 7608)	Complex PCI (*n* = 3804)	Non-Complex PCI (*n* = 3804)	*p*-Value
**Angiographic characteristics**				
Number of diseased vessels	1.6 ± 0.8	2.2 ± 0.6	1.0 ± 0.1	<0.001
Number of vessels treated	1.3 ± 0.5	1.6 ± 0.6	1.0 ± 0.1	<0.001
Number of lesions treated	1.2 ± 0.4	1.5 ± 0.5	1.0 ± 0.1	<0.001
Target vessels				
Left main	247 (3.2)	247 (6.5)	0 (0.0)	<0.001
Left anterior descending	4558 (59.9)	2370 (62.3)	2188 (57.5)	<0.001
Left circumflex	1864 (24.5)	1274 (33.5)	590 (15.5)	<0.001
Right coronary artery	2902 (38.1)	1854 (48.7)	1048 (27.5)	<0.001
Graft	5 (0.1)	2 (0.1)	3 (0.1)	0.655
Bifurcation	324 (4.3)	199 (5.2)	125 (3.3)	<0.001
**Procedural characteristics**				
Total stent length, mm	32.4 ± 19.5	40.9 ± 23.4	23.9 ± 8.4	<0.001
Total stent number	1.5 ± 0.8	1.9 ± 1.0	1.2 ± 0.4	<0.001
Mean stent diameter, mm	3.2 ± 0.4	3.1 ± 0.4	3.2 ± 0.4	<0.001
Type of stent implanted				
BMS	337 (4.4)	163 (4.3)	174 (4.6)	0.778
First-generation DES	1691 (22.2)	847 (22.3)	844 (22.2)	
Second-generation DES	5165 (67.9)	2586 (68.0)	2579 (67.8)	
Others	415 (5.5)	208 (5.5)	207 (5.4)	
Femoral access	8394 (81.3)	4981 (81.1)	3413 (81.6)	0.555
GP IIb/IIIa inhibitor	3231 (31.3)	1937 (31.5)	1294 (30.9)	0.528
Thrombolysis infusion	228 (2.2)	125 (2.0)	103 (2.5)	0.167
ECMO/IABP	236 (3.1)	121 (3.2)	115 (3.0)	0.677

Data are presented as the *n* (%) for categorical variables and as the mean ± standard deviation for continuous variables. PCI indicates percutaneous coronary intervention. PS—propensity score; BMS—bare metal stent; DES—drug eluting stent; GP—glycoprotein; ECMO—extracorporeal membrane oxygenation; IABP—intra-aortic balloon pumping.

**Table 3:** Impacts of each component of complex PCI or severe comorbidities on the risk of MACE in all AMI patients.

	MACE ^‡^ (*n* = 3320)	Without MACE ^‡^ (*n* = 7009)	Unadjusted		Multivariable-Adjusted		Propensity Score Matched	
			HR * (95% CI)	*p*-Value ^†^	HR (95% CI)	*p*-Value ^†^	HR * (95% CI)	*p*-Value ^†^
**Any of complex PCI components below**	2322 (69.9)	3822 (54.5)	1.72 (1.60–1.85)	<0.001	1.46 (1.35–1.57)	<0.001	1.50 (1.38–1.62)	<0.001
Left main as target lesion	184 (5.5)	248 (3.5)	1.70 (1.46–1.97)	<0.001	1.14 (0.98–1.33)	0.097	1.50 (1.20–1.87)	<0.001
Bifurcation with two stents (non-LM)	70 (2.1)	95 (1.4)	1.38 (1.09–1.75)	0.007	1.25 (0.99–1.59)	0.061	1.31 (0.94–1.81)	0.108
Multivessel PCI	2238 (67.4)	3617 (51.6)	1.71 (1.59–1.84)	<0.001	1.46 (1.35–1.57)	<0.001	1.48 (1.37–1.61)	<0.001
Long stenting (>60 mm)	184 (5.5)	276 (3.9)	1.36 (1.17–1.58)	<0.001	1.17 (1.00–1.36)	0.044	1.49 (1.22–1.83)	<0.001
Restenosis lesion	73 (2.2)	96 (1.4)	1.69 (1.34–2.13)	<0.001	1.44 (1.11–1.87)	0.006	1.92 (1.40–2.62)	<0.001
CTO lesion	196 (5.9)	321 (4.6)	1.22 (1.05–1.41)	0.007	1.20 (1.04–1.39)	0.013	1.34 (1.11–1.62)	0.002
At least 3 lesions treated	503 (15.2)	804 (11.5)	1.21 (1.10–1.33)	<0.001	1.15 (1.04–1.26)	0.006	1.18 (1.04–1.34)	0.009
At least 3 stents implanted	625 (18.8)	919 (13.1)	1.34 (1.23–1.46)	<0.001	1.20 (1.10–1.31)	<0.001	1.30 (1.16–1.47)	<0.001
**Components of severe comorbidities**								
Old age ≥ 75	1104 (33.3)	1175 (16.8)	2.31 (2.15–2.48)	<0.001	1.67 (1.54–1.81)	<0.001	2.48 (2.28–2.70)	<0.001
DM with insulin treatment	102 (3.1)	106 (1.5)	1.80 (1.47–2.19)	<0.001	1.22 (1.00–1.50)	0.055	1.29 (0.94–1.78)	0.113
Prior MI	171 (5.2)	204 (2.9)	1.58 (1.35–1.84)	<0.001	1.14 (0.95–1.37)	0.151	1.59 (1.32–1.92)	<0.001
Prior CABG	25 (0.8)	22 (0.3)	2.04 (1.38–3.02)	<0.001	1.32 (0.89–1.96)	0.171	2.30 (1.47–3.62)	<0.001
Established vascular disease	330 (9.9)	469 (6.7)	1.50 (1.34–1.68)	<0.001	1.54 (1.03–2.30)	0.033	1.53 (1.33–1.77)	<0.001
AF	143 (4.3)	171 (2.4)	1.56 (1.32–1.84)	<0.001	1.08 (0.91–1.28)	0.367	1.48 (1.18–1.85)	0.001
CKD (eGFR ≤30)	384 (11.6)	238 (3.4)	3.32 (2.99–3.70)	<0.001	1.60 (1.42–1.79)	<0.001	3.35 (2.90–3.88)	<0.001
Severe LV dysfunction (EF ≤ 35%)	378 (11.4)	356 (5.1)	2.20 (1.98–2.45)	<0.001	1.23 (1.10–1.37)	<0.001	1.94 (1.66–2.28)	<0.001
Cardiogenic shock	658 (19.8)	587 (8.4)	2.71 (2.49–2.95)	<0.001	1.21 (1.06–1.38)	0.006	2.56 (2.27–2.87)	<0.001
ECMO/IABP	306 (9.2)	132 (1.9)	4.67 (4.15–5.25)	<0.001	1.65 (1.44–1.89)	<0.001	4.46 (3.62–5.49)	<0.001

Values are number of events (%) unless otherwise indicated. * Cox regression with robust sandwich variance estimator. ^†^ *p* value from univariate Cox regression. ^‡^ Defined as the composite of cardiac death, myocardial infarction, definite or probable stent thrombosis or revascularization. HR indicates hazard ratio; PS—propensity score; CI—confidence interval; MACE—major adverse cardiac events; PCI—percutaneous coronary intervention; AMI—acute myocardial infarction; LM—left main; CTO—chronic total occlusion; DM—diabetes mellitus; MI—myocardial infarction; CABG—coronary artery bypass graft; AF—atrial fibrillation; CKD—chronic kidney disease; eGFR—estimated glomerular filtration rate; EF—ejection fraction; LV—left ventricle; ECMO—extracorporeal membrane oxygenation; IABP—intra-aortic balloon pumping.

**Table 4:** Ischemic and bleeding outcomes in all AMI patients according to PCI complexity.

	Complex PCI (*n* = 6144)	Non-Complex PCI (*n* = 4185)	Unadjusted		Multivariable-Adjusted		Propensity Score Matched	
			HR * (95% CI)	*p*-Value ^†^	HR (95% CI)	*p*-Value ^†^	HR * (95% CI)	*p*-Value ^†^
**Ischemic endpoints**								
MACE ^‡^	2322 (37.8)	998 (23.8)	1.72 (1.60–1.85)	<0.001	1.46 (1.35–1.57)	<0.001	1.50 (1.38–1.62)	<0.001
Cardiac death	1350 (22.0)	580 (13.9)	1.60 (1.45–1.76)	<0.001	1.19 (1.07–1.31)	0.001	1.16 (1.04–1.29)	0.007
MI	365 (5.9)	165 (3.9)	1.56 (1.29–1.87)	<0.001	1.44 (1.19–1.74)	<0.001	1.51 (1.23–1.86)	<0.001
Definite or probable ST	119 (1.9)	49 (1.2)	1.69 (1.21–2.35)	0.002	1.70 (1.20–2.40)	0.003	1.96 (1.36–2.83)	<0.001
Revascularization	1061 (17.3)	431 (10.3)	1.83 (1.63–2.04)	<0.001	1.82 (1.62–2.04)	<0.001	1.87 (1.65–2.12)	<0.001
All-cause death	1689 (27.5)	788 (18.8)	1.48 (1.36–1.61)	<0.001	1.11 (1.02–1.21)	0.019	1.06 (0.96–1.16)	0.248
Ischemic stroke	186 (3.0)	100 (2.4)	1.29 (1.01–1.64)	0.042	1.10 (0.86–1.42)	0.442	1.16 (0.88–1.53)	0.294
Target vessel revascularization	497 (8.1)	266 (6.4)	1.31 (1.13–1.52)	<0.001	1.27 (1.09–1.48)	0.002	1.29 (1.09–1.53)	0.003
Target lesion revascularization	354 (5.8)	222 (5.3)	1.11 (0.94–1.31)	0.224	1.07 (0.90–1.28)	0.427	1.12 (0.92–1.35)	0.250
**Bleeding endpoints**								
BARC 2, 3 or 5	735 (12.0)	408 (9.7)	1.26 (1.12–1.42)	<0.001	1.09 (0.96–1.23)	0.184	1.08 (0.94–1.24)	0.273
BARC 3 or 5	499 (8.1)	254 (6.1)	1.37 (1.18–1.60)	<0.001	1.14 (0.98–1.33)	0.099	1.14 (0.95–1.35)	0.153
Any bleeding	912 (14.8)	552 (13.2)	1.16 (1.04–1.29)	0.007	1.06 (0.95–1.19)	0.277	1.05 (0.93–1.18)	0.455

Values are number of events (%) unless otherwise indicated. * Cox regression with robust sandwich variance estimator. ^†^ *p* value from univariate Cox regression. ^‡^ Defined as the composite of cardiac death, myocardial infarction, definite or probable stent thrombosis or revascularization. AMI indicates acute myocardial infarction; PCI—percutaneous coronary intervention; HR—hazard ratio; PS—propensity score; CI—confidence interval; MACE—major adverse cardiac events; MI—myocardial infarction; ST—stent thrombosis; BARC—bleeding academic research consortium.

**Table 5:** Ischemic and bleeding outcomes in patients with severe comorbidities or without severe comorbidities according to procedure complexity (propensity score matched cohort).

	Patients without Severe Comorbidities (*n* = 4589)				Patients with Severe Comorbidities (*n* = 3019)			
	Complex PCI	Non-Complex PCI	Univariate HR * (95% CI)	*p*-Value ^†^	Complex PCI	Non-Complex PCI	Univariate HR * (95% CI)	*p*-Value ^†^
**Ischemic endpoints**								
MACE ^‡^	618 (26.9)	364 (15.9)	1.82 (1.60–2.08)	<0.001	681 (45.3)	564 (37.2)	1.29 (1.16–1.44)	<0.001
Cardiac death	155 (6.7)	124 (5.4)	1.24 (0.98–1.57)	0.073	495 (33.0)	433 (28.6)	1.14 (1.01–1.30)	0.034
MI	129 (5.6)	60 (2.6)	2.16 (1.59–2.94)	<0.001	93 (6.2)	88 (5.8)	1.07 (0.80–1.43)	0.635
Definite or probable ST	45 (2.0)	21 (0.9)	2.14 (1.27–3.59)	0.004	39 (2.6)	22 (1.5)	1.81 (1.08–3.03)	0.024
Revascularization	462 (20.1)	250 (10.9)	1.97 (1.68–2.30)	<0.001	222 (14.8)	140 (9.2)	1.70 (1.37–2.10)	<0.001
All-cause death	209 (9.1)	185 (8.1)	1.12 (0.92–1.36)	0.271	594 (39.5)	570 (37.6)	1.04 (0.93–1.17)	0.443
Ischemic stroke	50 (2.2)	37 (1.6)	1.34 (0.88–2.04)	0.18	57 (3.8)	55 (3.6)	1.05 (0.73–1.52)	0.79
Target vessel revascularization	200 (8.7)	150 (6.6)	1.34 (1.08–1.65)	0.007	104 (6.9)	87 (5.7)	1.21 (0.91–1.61)	0.194
Target lesion revascularization	144 (6.3)	127 (5.6)	1.13 (0.89–1.43)	0.333	81 (5.4)	74 (4.9)	1.10 (0.80–1.51)	0.551
**Bleeding endpoints**								
BARC 2, 3 or 5	182 (7.9)	166 (7.3)	1.09 (0.89–1.34)	0.407	228 (15.2)	216 (14.3)	1.09 (0.90–1.31)	0.387
BARC 3 or 5	109 (4.7)	83 (3.6)	1.31 (0.99–1.73)	0.062	164 (10.9)	159 (10.5)	1.06 (0.85–1.32)	0.619
Any bleeding	266 (11.6)	256 (11.2)	1.03 (0.87–1.22)	0.716	267 (17.8)	256 (16.9)	1.07 (0.91–1.27)	0.413

Values are number of events (%) unless otherwise indicated. * Cox regression with robust sandwich variance estimator. ^†^ *p* value from univariate Cox regression. ^‡^ Defined as the composite of cardiac death, myocardial infarction, definite or probable stent thrombosis or revascularization. HR indicates hazard ratio; CI—confidence interval; PCI—percutaneous coronary intervention; MACE—major adverse cardiac events; ST—stent thrombosis; BARC—bleeding academic research consortium.

## Data Availability

The datasets used and/or analyzed during the current study are available from the corresponding author on reasonable request.

## Supplementary Materials

The following supporting information can be downloaded at: https://www.mdpi.com/article/10.3390/jcm11164853/s1, Table S1: Ischemic and Bleeding Outcomes in AMI Patients According to PCI Complexity (three-month landmark analysis); Table S2: Ischemic and Bleeding Outcomes in AMI Patients According to PCI Complexity beyond three months; Table S3: Ischemic and Bleeding Outcomes in AMI Patients According to PCI Complexity beyond one year. Table S4. In-hospital bleeding outcomes in patients with acute myocardial infarction.
