# Supplementary material for: Independent Clinical Impacts of Procedural Complexity on Ischemic and Bleeding Events in Patients with Acute Myocardial Infarction: Long-Term Clinical Study
Source: J Clin Med. 2022 Aug 18;11(16):4853. doi: 10.3390/jcm11164853 (PMC9410511; doi:10.3390/jcm11164853)
Supplement: Supplementary file 1 [file jcm-11-04853-s001.zip › jcm-1828796-supplementary.pdf]

Supplementary Materials

Table S1. Ischemic and Bleeding Outcomes in AMI Patients According to PCI Complexity (three-month landmark analysis).

(A) ≤ three-months.

	Complex PCI	Non-complex PCI	Unadjusted		Multivariable-adjusted		Propensity score matched	
			HR* (95% CI)	p-value†	HR (95% CI)	p-value†	HR* (95% CI)	p-value†
Ischemic endpoints								
MACE‡	288 (7.8)	203 (5.5)	1.43(1.20-1.70)	<0.001	1.45(1.23-1.71)	<0.001	1.43(1.20-1.70)	<0.001
Cardiac death	200 (5.4)	180 (4.9)	1.11(0.91-1.35)	0.293	1.13(0.94-1.35)	0.206	1.11(0.91-1.35)	0.293
MI	46 (1.3)	19 (0.5)	2.43(1.43-4.12)	0.001	2.46(1.48-4.09)	<0.001	2.43(1.43-4.12)	0.001
Definite or probable ST	42 (1.2)	15 (0.4)	2.81(1.57-5.02)	<0.001	2.83(1.63-4.92)	<0.001	2.81(1.57-5.02)	<0.001
Revascularization	64 (1.8)	19 (0.5)	3.39(2.03-5.68)	<0.001	3.05(1.90-4.91)	<0.001	3.39(2.03-5.68)	<0.001
All-cause death	213 (5.7)	208 (5.6)	1.03(0.85-1.24)	0.78	1.03(0.87-1.22)	0.733	1.03(0.85-1.24)	0.78
Ischemic stroke	34 (1.0)	18 (0.5)	1.90(1.07-3.36)	0.028	1.60(0.94-2.73)	0.084	1.90(1.07-3.36)	0.028
Target vessel revascularization	27 (0.8)	10 (0.3)	2.71(1.31-5.61)	0.007	2.50(1.25-4.99)	0.009	2.71(1.31-5.61)	0.007
Target lesion revascularization	28 (0.8)	9 (0.3)	3.12(1.47-6.63)	0.003	2.91(1.42-5.98)	0.004	3.12(1.47-6.63)	0.003
Bleeding endpoints								
BARC 2, 3, or 5	182 (5.2)	142 (4.0)	1.29(1.04-1.61)	0.024	1.26(1.03-1.54)	0.025	1.29(1.04-1.61)	0.024
BARC 3, or 5	157 (4.4)	121 (3.4)	1.31(1.03-1.66)	0.028	1.27(1.02-1.57)	0.033	1.31(1.03-1.66)	0.028
Any bleeding	76 (2.2)	79 (2.2)	0.96(0.70-1.33)	0.826	0.97(0.73-1.29)	0.845	0.96(0.70-1.33)	0.826

Values are number of events (%) unless otherwise indicated. *Cox regression with robust sandwich variance estimator. †p value from univariate Cox regression. ‡Defined as the composite of cardiac death, myocardial infarction, definite or probable stent thrombosis or revascularization. HR indicates hazard ratio; PS, propensity score; CI, confidence interval; MACE, major adverse cardiac events; MI, myocardial infarction; ST, stent thrombosis; BARC, bleeding academic research consortium.

(B) > three-months, ≤ one-year.

	Complex PCI	Non-complex PCI	Unadjusted		Multivariable-adjusted		Propensity score matched	
			HR* (95% CI)	p-value†	HR (95% CI)	p-value†	HR* (95% CI)	p-value†
Ischemic endpoints								
MACE‡	293 (8.6)	180 (5.2)	1.69(1.40-2.03)	<0.001	1.63(1.38-1.94)	<0.001	1.69(1.40-2.03)	<0.001
Cardiac death	75 (2.1)	64 (1.8)	1.18(0.84-1.64)	0.339	1.11(0.83-1.49)	0.474	1.18(0.84-1.64)	0.339
MI	35 (1.0)	22 (0.6)	1.61(0.94-2.75)	0.082	1.75(1.07-2.85)	0.025	1.61(0.94-2.75)	0.082
Definite or probable ST	5 (0.1)	2 (0.1)	2.52(0.49-13.01)	0.268	2.50(0.49-12.62)	0.269	2.52(0.49-13.01)	0.268
Revascularization	221 (6.4)	112 (3.2)	2.04(1.63-2.56)	<0.001	2.01(1.62-2.47)	<0.001	2.04(1.63-2.56)	<0.001
All-cause death	97 (2.8)	89 (2.5)	1.09(0.82-1.46)	0.54	1.10(0.85-1.41)	0.464	1.09(0.82-1.46)	0.54
Ischemic stroke	10 (0.3)	14 (0.4)	0.72(0.32-1.62)	0.428	0.69(0.34-1.40)	0.3	0.72(0.32-1.62)	0.428
Target vessel revascularization	92 (2.6)	71 (2.0)	1.31(0.96-1.79)	0.088	1.25(0.94-1.66)	0.118	1.31(0.96-1.79)	0.088
Target lesion revascularization	59 (1.7)	56 (1.6)	1.06(0.74-1.53)	0.736	1.03(0.74-1.44)	0.861	1.06(0.74-1.53)	0.736
Bleeding endpoints								
BARC 2, 3, or 5	63 (1.9)	75 (2.2)	0.85(0.61-1.19)	0.355	0.83(0.62-1.12)	0.22	0.85(0.61-1.19)	0.355
BARC 3, or 5	34 (1.0)	38 (1.1)	0.91(0.57-1.44)	0.682	0.85(0.56-1.30)	0.449	0.91(0.57-1.44)	0.682
Any bleeding	109 (3.3)	126 (3.7)	0.88(0.68-1.13)	0.307	0.86(0.68-1.08)	0.188	0.88(0.68-1.13)	0.307

Values are number of events (%) unless otherwise indicated. *Cox regression with robust sandwich variance estimator. †p value from univariate Cox regression. ‡Defined as the composite of cardiac death, myocardial infarction, definite or probable stent thrombosis or revascularization. HR indicates hazard ratio; PS, propensity score; CI, confidence interval; MACE, major adverse cardiac events; MI, myocardial infarction; ST, stent thrombosis; BARC, bleeding academic research consortium.

Table S2. Ischemic and Bleeding Outcomes in AMI Patients According to PCI Complexity beyond Three Months.

	Complex PCI	Non-complex PCI	Unadjusted	
			HR* (95% CI)	p-value†
Ischemic endpoints				
MACE‡	1011 (29.6)	725 (20.8)	1.51(1.38-1.66)	<0.001
Cardiac death	450 (12.8)	377 (10.7)	1.18(1.04-1.34)	0.013
MI	176 (5.1)	129 (3.7)	1.38(1.10-1.73)	0.006
Definite or probable ST	42 (1.2)	28 (0.8)	1.50(0.93-2.43)	0.095
Revascularization	620 (18.0)	371 (10.6)	1.79(1.57-2.04)	<0.001
All-cause death	590 (16.8)	547 (15.6)	1.07(0.96-1.19)	0.247
Ischemic stroke	73 (2.1)	74 (2.1)	0.98(0.71-1.36)	0.912
Target vessel revascularization	277 (8.0)	227 (6.5)	1.23(1.03-1.47)	0.021
Target lesion revascularization	197 (5.7)	192 (5.5)	1.02(0.84-1.25)	0.809
Bleeding endpoints				
BARC 2, 3, or 5	228 (6.8)	240 (7.1)	0.96(0.80-1.14)	0.614
BARC 3, or 5	116 (3.4)	121 (3.5)	0.96(0.75-1.25)	0.78
Any bleeding	321 (9.7)	333 (9.9)	0.97(0.83-1.13)	0.668

Values are number of events (%) unless otherwise indicated. *Cox regression with robust sandwich variance estimator. †p value from univariate Cox regression. ‡Defined as the composite of cardiac death, myocardial infarction, definite or probable stent thrombosis or revascularization. HR indicates hazard ratio; PS, propensity score; CI, confidence interval; MACE, major adverse cardiac events; MI, myocardial infarction; ST, stent thrombosis; BARC, bleeding academic research consortium.

Table S3. Ischemic and Bleeding Outcomes in AMI Patients According to PCI Complexity beyond One Year.

	Complex PCI	Non-complex PCI	Unadjusted		Multivariable-adjusted		Propensity score matched	
			HR* (95% CI)	p-value†	HR (95% CI)	p-value†	HR* (95% CI)	p-value†
Ischemic endpoints								
MACE‡	1262 (26.5)	588 (16.6)	1.64(1.49-1.81)	<0.001	1.37(1.24-1.52)	<0.001	1.45(1.30-1.62)	<0.001
Cardiac death	764 (14.7)	324 (8.8)	1.61(1.41-1.84)	<0.001	1.20(1.05-1.37)	0.009	1.18(1.02-1.36)	0.024
MI	222 (4.3)	122 (3.3)	1.28(1.03-1.60)	0.028	1.18(0.94-1.49)	0.152	1.33(1.03-1.71)	0.026
Definite or probable ST	51 (1.0)	29 (0.8)	1.23(0.78-1.94)	0.374	1.18(0.74-1.88)	0.498	1.43(0.86-2.36)	0.166
Revascularization	627 (13.1)	284 (8.0)	1.67(1.45-1.92)	<0.001	1.63(1.41-1.89)	<0.001	1.68(1.44-1.96)	<0.001
All-cause death	1010 (19.4)	477 (12.9)	1.45(1.30-1.62)	<0.001	1.09(0.97-1.22)	0.13	1.06(0.94-1.20)	0.315
Ischemic stroke	116 (2.2)	65 (1.8)	1.23(0.91-1.67)	0.179	1.05(0.77-1.44)	0.757	1.04(0.73-1.49)	0.818
Target vessel revascularization	315 (6.2)	174 (4.8)	1.27(1.05-1.53)	0.012	1.20(0.99-1.45)	0.061	1.20(0.98-1.49)	0.084
Target lesion revascularization	226 (4.4)	149 (4.1)	1.05(0.85-1.29)	0.652	0.98(0.79-1.22)	0.858	1.02(0.80-1.29)	0.897
Bleeding endpoints								
BARC 2, 3, or 5	301 (6.1)	176 (5.0)	1.20(1.00-1.45)	0.054	1.02(0.84-1.23)	0.854	0.99(0.80-1.23)	0.961
BARC 3, or 5	155 (3.1)	86 (2.4)	1.27(0.98-1.65)	0.076	1.03(0.79-1.36)	0.818	0.99(0.73-1.35)	0.948
Any bleeding	376 (7.8)	222 (6.5)	1.19(1.00-1.40)	0.044	1.07(0.90-1.27)	0.457	1.02(0.84-1.23)	0.855

Values are number of events (%) unless otherwise indicated. *Cox regression with robust sandwich variance estimator. †p value from univariate Cox regression. ‡Defined as the composite of cardiac death, myocardial infarction, definite or probable stent thrombosis or revascularization. HR indicates hazard ratio; PS, propensity score; CI, confidence interval; MACE, major adverse cardiac events; MI, myocardial infarction; ST, stent thrombosis; BARC, bleeding academic research consortium.

Table S4. In-hospital bleeding outcomes in patients with acute myocardial infarction

			Original cohort		PS-matched cohort	
	No in-hospital bleeding (N=9943)	In-hospital bleeding (N=386)	HR (95% CI)	p-value†	HR (95% CI)	p-value†
Complex PCI	5882 (59.2)	262 (67.9)	1.46 (1.18,1.8)	< 0.001	1.28 (1-1.63)	0.046
Age	63.3 ± 12.7	69.2 ± 12.1	1.04 (1.03,1.05)	< 0.001		
Female	2763 (27.8)	170 (44.0)	2.07 (1.69,2.53)	< 0.001		
DM	3622 (36.4)	166 (43.0)	1.32 (1.08,1.62)	0.006		
eGFR <30	565 (5.7)	57 (14.8)	3.07 (2.31,4.07)	< 0.001		
History of stroke	704 (7.1)	46 (11.9)	1.79 (1.32,2.44)	< 0.001		
LVEF ≤35%	677 (6.8)	57 (14.8)	2.38 (1.8,3.16)	< 0.001		
Femoral access	8085 (81.3)	309 (80.1)	0.9 (0.7,1.15)	0.399		
GP IIB/IIIA inhibitor	3096 (31.1)	135 (35.0)	1.17 (0.95,1.45)	0.137		
Thrombolysis infusion	218 (2.2)	10(2.6)	1.12 (0.6,2.11)	0.714		
ECMO or IABP	372 (3.7)	66 (17.1)	6.2 (4.76,8.1)	< 0.001		

Values are number of events (%) unless otherwise indicated. †p value from univariate Cox regression. GP IIB/IIIIA inhibitor includes abciximab and tirofiban, eptifibatide, and agatroban. Thrombolysis includes urokinase, actilyse, and metalyse. HR indicates hazard ratio; CI, confidence interval; DM, diabetes mellitus; eGFR, estimated glomerular filtration rate; LVEF, left ventricle ejection fraction; GP, glycoprotein; ECMO, extracorporeal membrane oxygenation; IABP, intra-aortic balloon pumping.
